# Coronavirus disease 2019 pandemic two years later… What’s next?

**DOI:** 10.3325/cmj.2022.63.1

**Published:** 2022-02

**Authors:** Rok Čivljak, Alemka Markotić

**Affiliations:** 1 Dr. Fran Mihaljević University Hospital for Infectious Diseases, Zagreb, Croatia; 2 University of Zagreb School of Medicine, Zagreb, Croatia *rok.civljak@bfm.hr*; 3 Nursing Studies, School of Medicine, Catholic University of Croatia, Zagreb, Croatia; 4 University of Rijeka School of Medicine, Rijeka, Croatia

## Looking back on the onset of the pandemic

Exactly two years ago, we published an editorial in the *Croatian Medical Journal* discussing a global threat from a new coronavirus epidemic caused by the SARS-CoV-2 virus, which began in China at the end of 2019 and soon spread around the world (1). The first case of COVID-19 in Croatia was recorded on February 25, 2020, while the editorial was being written. On that occasion, we warned of the danger of the rapid spread and pathogenic potential of the novel coronavirus, the need for the constant monitoring of respiratory pathogens with high epidemic potential, as well as of the importance of developing vaccines and antiviral drugs rapidly to combat this and similar viruses.

The epidemic came to Croatia from Italy and other neighboring countries, indicating the significance of the spreading of infectious diseases through migrations and travels. This is why it is of utmost importance to cooperate internationally and undertake the collaborative measures by the governments of neighboring countries for combating epidemics/pandemics of infectious diseases (2).

Today, two years later, an unpleasant and unexpected scenario has unfolded that we could not have imagined, but the question remains: What’s next?

To date, the COVID-19 pandemic has posed a significant global threat of great epidemic magnitude, resulting in over 424 million confirmed cases and more than 5.8 million deaths worldwide (3).

## Epidemic pattern of COVID-19 in Croatia

The numbers of the newly infected and fatal cases during the COVID-19 pandemic varied in different parts of the world, and also within individual countries. Europe was hit by five waves of the pandemic. The Omicron variant of the SARS-CoV-2 virus brought the greatest number of the infected and ill, but the most severe cases were caused by the delta variant (4).

At the beginning, Croatia managed the situation very successfully, promptly implementing the measures to combat the spread of the epidemic within its borders. The epidemic in Croatia was declared on March 11, 2020, the same day when the World Health Organization declared a pandemic. On March 19, 2020, the Civil Protection Headquarters of the Republic of Croatia imposed a lockdown, which abolished all public gatherings and enacted strict anti-epidemic measures to prevent the spread of SARS-CoV-2.

At the beginning of the pandemic, Croatia had one of the highest COVID-19 Stringency Index values and now is one of the most liberal countries in the EU with regard to the stringency of anti-epidemic measures. Due to strict measures during the first wave, Croatia was one of the countries with the lowest daily number of new confirmed COVID-19 cases and cumulative confirmed COVID-19 deaths per capita (5). The incidence of COVID-19 cases was not even increased by another natural disaster afflicting Croatia: two earthquakes hit Zagreb and destroyed a large number of buildings. Fortunately, the number of human casualties was small, but a lot of people needed to be evacuated from their homes (6).

However, in late 2020, during the second wave of the pandemic, Croatia had one of the highest incidences of daily new confirmed COVID-19 cases per capita in Europe. In February 2022, it temporarily had the highest 14-day death rate per capita in the EU/EEA. Sadly, the vaccination rate in Croatia is one of the lowest in the EU, which, along with high prevalence of certain chronic diseases in Croatian population and unfavorable demographic picture, is one of the factors affecting the high incidence and death rate in our country. Up to now, 1 053 568 cases have been registered in Croatia, with over 15 000 deaths (5). However, the final statistics could be comparable among different countries when all data are recorded, presented, and analyzed systematically. In addition, future comparisons will need to take into account not just numbers, but all the specificities of health status and systems in individual countries, as well as elements affecting morbidity and mortality.

## What have we learnt so far about diagnostics, clinical presentation, and treatment of COVID-19?

Early identification of SARS-CoV-2 was of paramount importance for the fight against the pandemic, including development of diagnostic tests. The first real-time RT-PCR test was developed in January 2020 (7), and in a week its use was established at the University Hospital for Infectious Diseases in Zagreb, almost a month before the first COVID-19 case was detected. Consequently, serology tests and point-of-care, rapid antigenic tests were developed and implemented in the diagnostics of acute cases. They were also applied in the monitoring of antibody response during illness but also after vaccination (8,9).

It was noticed early on that the virus has a great ability to mutate, and several variants were detected in 2020 (10). So far, there have been five dominant variants of SARS-CoV-2 spreading among global populations: the Alpha variant (B.1.1.7, formerly called the UK variant), the Beta variant (B.1.351, formerly called the South Africa variant), the Gamma variant (P.1, formerly called the Brazil variant), the Delta variant (B.1.617.2, formerly called the India variant), and the Omicron variant (B.1.1.529) (11).

Clinical manifestations of COVID-19 range from asymptomatic and mild disease to critical illness. At the beginning of the pandemic, the percentage of symptomatic and severe cases was higher than later in the pandemic (12,13).

Hospitalization and crude mortality rates increase with age, and patients with underlying comorbidities, such as hypertension, diabetes, cardiovascular diseases, chronic obstructive pulmonary disease, obesity, immunodeficiency and other risk factors, are at a higher risk of progressing to severe COVID-19 and fatal outcome (13).

With the progression of the pandemic and growing numbers of patients, it was seen that COVID-19 is not only an acute disease but that in some patients it can also have a protracted or even relapsing form, known as post-COVID syndrome, post-acute sequelae of SARS-CoV-2 infection, or long COVID. Post-COVID-19 conditions occur in individuals with a history of SARS-CoV-2 infection, usually three months from the onset of symptoms, last for at least two months, and cannot be explained by an alternative diagnosis. Common symptoms include fatigue, shortness of breath, cognitive dysfunction, and others, and generally affect everyday functioning. Symptoms may also fluctuate or relapse over time (14). Long-term consequences from COVID-19 can especially be expected among patients treated in an intensive care unit. A recently published study showed that three-quarters of intensive care unit COVID-19 survivors had physical symptoms at one year, including physical weakness, joint symptoms, and myalgia. Mental symptoms were reported by 26% and cognitive symptoms by 16% of the survivors. These symptoms are consistent with post-intensive care syndrome and need to be further investigated after recovery from COVID-19 (15).

Symptomatic and supportive therapies, particularly oxygen replacement therapy, are still the basis for the treatment of all forms of COVID-19. The first attempts at the etiological treatment at the beginning of the pandemic, such as the treatment with chloroquine or hydroxychloroquine, lopinavir/ritonavir, favipiravir, azithromycin, interferon etc, did not yield significant results in terms of cure or the prevention of progression to the severe form. At the beginning, studies were limited to a small number of participants but later the sample sizes increased, yielding statistically significant results.

WHO has launched a non-blinded clinical trial (SOLIDARITY) to evaluate candidate treatments vs standard of care in 18 countries worldwide (16). Although this study did not demonstrate benefit from the use of remdesivir, a drug that seemed highly promising in the beginning, other studies demonstrated that it shortened the duration of the disease and reduced mortality (17,18). The study was recently expanded (the Solidarity PLUS Trial) to include three new drugs – artesunate, infliximab, and imatinib – thus empowering local researchers and providing them with opportunities to contribute their expertise and resources to global research (19).

A large UK-based, controlled, open-label trial comparing a range of possible treatments for patients hospitalized with Covid-19 (RECOVERY Study) found that dexamethasone reduced deaths among those who were receiving either invasive mechanical ventilation or oxygen alone but not among those receiving no respiratory support (20). Moreover, a large international trial, REMAP-CAP, found that drugs that block a key immune protein – the interleukin-6 receptor – can reduce the risk of death among critically ill COVID-19 patients (21).

Today, the guidelines for COVID-19 treatment include modified antiviral drugs (remdesivir, molnupiravir, nirmatrelvir/ritonavir), systemic corticosteroids (dexamethasone, metylprednisolone), immunomodulatory drugs (tocilizumab, sarilumab, baricitinib, anakinra), monoclonal antibodies (casirivimab/imdevimab, bamlanivimab/etesevimab, sotrovimab, tixagevimab/cilgavimab, regdanvimab), and COVID-19 convalescent plasma (22,23).

Antibiotics are indicated for the treatment of secondary bacterial complications, which are extremely rare in COVID-19, except as nosocomial infections encountered in the intensive care units. Although azithromycin is reported to have antiviral and anti-inflammatory/immunomodulatory properties, studies have not confirmed a clinical benefit in the treatment of COVID-19, and therefore its use is unwarranted (24,25), as is the use of the anti-parasitic drug ivermectin (26).

The goal of the current clinical approach to the care of COVID-19 patients is the early treatment of patients with mild-to-moderate COVID-19 infection at high risk of progressing to severe COVID-19 and hospitalization with ritonavir-boosted nirmatrelvir, sotrovimab, remdesivir, or molnupiravir. In advanced disease, the emphasis is on hospital care for those who have developed respiratory insufficiency and intensive care, if necessary, including immunomodulatory treatment with anakinra, a recombinant human interleukin-1 receptor antagonist, dexamethasone with/without remdesivir, or the addition of a second immunomodulatory drug, eg, monoclonal antibodies that inhibit the pro-inflammatory action of interleukin 6 (tocilizumab or sarilumab) or an orally-administered selective inhibitor of Janus kinases 1 and 2 (baricitinib) (22,23).

## Importance of newly introduced vaccines in treatment of COVID-19

Vaccines have aroused great hope as weapons for combating this pandemic. Shortly after virus sequencing, enormous resources and scientific potential were invested in research on the development of vaccines against SARS-CoV-2 (27). As a result, less than a year from the outbreak of the pandemic, the first vaccine obtained emergency use authorization in the USA and EU in December 2020. To date, five vaccines have been authorized for use in the European Union: two recombinant mRNA, two vector, and one protein vaccine. Other five vaccines are currently under rolling review by the EMA for marketing authorization (28).

WHO is one of the leaders of a global alliance known as COVAX, the vaccine pillar of the ACT-Accelerator collaboration, which is working to accelerate the development and manufacture of COVID-19 vaccines and ensure that all countries have fair and equitable access to them (29). With an efficiency of over 90%, vaccines helped arrest the progress of the pandemic during 2021. However, breakthrough cases began to appear, which indicated the insufficiency and brevity of the protection provided by the vaccines. Therefore, booster vaccines began to be administered to persons who had been vaccinated or had recovered from the virus. Nevertheless, with the appearance of the Omicron variant in November 2021, vaccination provided a weaker protective effect, even among those who had received three doses, although their rates of hospitalization and mortality were significantly lower (30). Certainly, new-generation, multivariant vaccines should be considered for the future development and production.

## Large-scale effects of the pandemic

In addition to a direct effect on morbidity and mortality, the COVID-19 pandemic has also caused a number of other changes in health care and society. Some studies have shown that the quality of the health care received by patients with other diseases, particularly non-communicable ones, has declined owing to the system’s focus on COVID-19. Moreover, changes in the organization of daily life due to anti-epidemic measures, such as the closing of schools, banning of gatherings, and limitation of cultural events, have led to a social depression whose effects will only be known in retrospect (31).

The recent mutation of SARS-CoV-2 to the milder but highly transmissible Omicron variant has caused an explosive increase in the numbers of infected persons but a small percentage of severe forms of the disease. There is hope that it could lead to the development of herd immunity and bring an end to the pandemic (32,33). Nevertheless, other scenarios are also possible, for which the health care system and society as a whole must be prepared in the event of a new escalation of the pandemic.

This editorial, like its predecessor two years ago, could be concluded with the same words: What’s next? Let us hope for a brighter future!
